# Modeling Glioblastoma for Translation: Strengths and Pitfalls of Preclinical Studies

**DOI:** 10.3390/biology14111490

**Published:** 2025-10-24

**Authors:** Concetta D’Antonio, Giovanna L. Liguori

**Affiliations:** Institute of Genetics and Biophysics (IGB) “Adriano Buzzati-Traverso”, National Research Council (CNR) of Italy, 80131 Naples, Italy; co.dantonio98@gmail.com

**Keywords:** glioblastoma, preclinical models, clinical translation, strengths and weaknesses analysis, organoids, microfluidics, bioprinting, GB-on-a-chip, xenografts, in silico modeling

## Abstract

**Simple Summary:**

Glioblastoma is the most aggressive brain tumor in adults, with very few treatment options and a poor survival rate. One of the biggest challenges in improving therapy is the use of reliable models that reflect the complexity of the disease to use in preclinical tests of new candidate drugs. This review presents the main research models to study glioblastoma, from standard cell lines to more advanced three-dimensional cellular and computational systems aiming to recreate the complexity of the tumor architecture inside the brain microenvironment. Animal models, including zebrafish, are also reviewed, as they provide important insights into how glioblastoma behaves in living organisms. This review offers an original perspective, examining the origins, objectives, strengths, weaknesses and clinical relevance of each model, and highlights how the ideal model system is a chimera. To increase knowledge of glioblastoma progression and chances of clinical success, preclinical data from a set of integrated technologies and multiple models tailored to the specific aims of this study must be carefully collected and compared.

**Abstract:**

Glioblastoma (GB) is an extremely aggressive tumor for which effective therapy is still in its infancy. Although several candidate therapeutics have been identified in functional preclinical assays, clinical trials have not supported their effectiveness in GB patients. The poor clinical efficacy of the treatments can be attributed to the insufficient mimicry of GB in patients by the preclinical models used. In this review article, we provide a comprehensive overview of the available GB preclinical models, which are classified according to their origin (animal or human), species, type and modeling strategy (two- or three-dimensional cell culture, in vivo grafting or in silico modeling). Moreover, the article compares developing cutting-edge technologies, including GB-derived organoids, bioprinting, microfluidic devices, and their multimodal integration in GB-on-chip systems, which aim to replicate the GB microenvironment with high precision. In silico and in vivo approaches are also reviewed, including zebrafish transplantation models. The costs, benefits, applications and clinical relevance of each model system and/or modeling strategy are discussed in detail and compared. We highlight that the most appropriate, or combination of, GB preclinical models must be selected (or even customized) based on the specific aims and constraints of each study. Finally, to improve the reliability and translational relevance of GB research, we propose a practical roadmap that addresses critical challenges in preclinical assay development, ranging from short-term adjustments to long-term strategic planning.

## 1. Introduction

**Figure 1:**
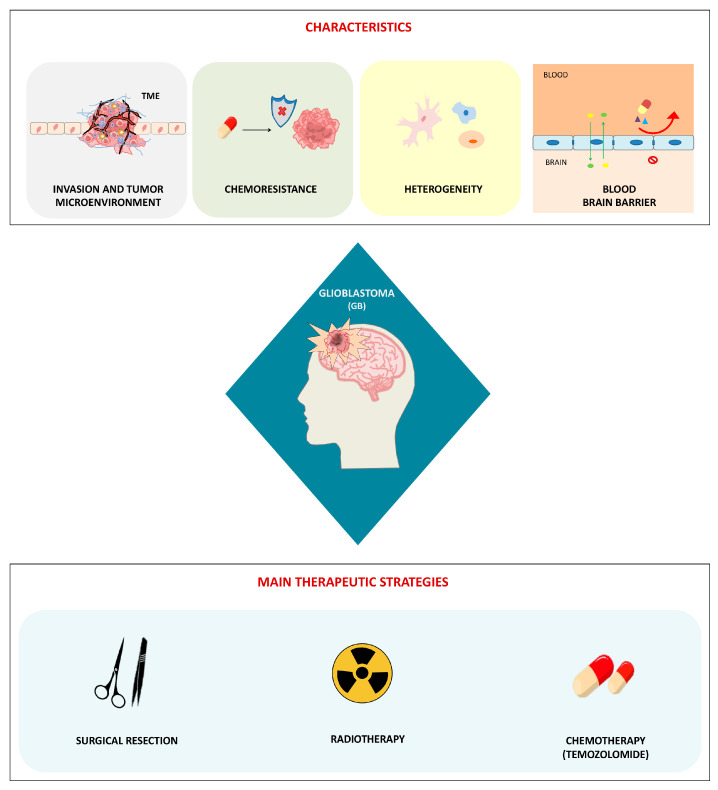
Schematic representation of the main hallmarks and current therapeutic strategies for glioblastoma. Glioblastoma (GB) is characterized by aggressive and invasive growth, strongly influenced by tumor microenvironment, high resistance to chemotherapeutic agents (chemoresistance), cellular heterogeneity and the presence of a highly selective blood–brain barrier that limits drug delivery to the tumor site. Standard therapies include surgical resection, radiotherapy and temozolomide-based chemotherapy. Despite this multimodal approach, tumor relapse remains common.

Glioblastoma (GB) is the most frequent and aggressive primary malignant tumor of the central nervous system in adults, classified as grade IV by the World Health Organization. Despite standard treatment, which includes surgical resection, followed by radiotherapy and concomitant and adjuvant temozolomide (TMZ) chemotherapy, median overall survival remains approximately with a prognosis of 12–15 months, and just 3–5% survival over 5 years [1,2,3]. The treatment of GB poses significant therapeutic challenges due to several factors: the tumor’s highly invasive nature, rapid development of resistance to radio- and chemotherapy, early recurrence, marked heterogeneity and the presence of the blood–brain barrier (BBB), which most pharmacological compounds are unable to penetrate [3,4]. GB cells are capable of invading healthy brain tissue by degrading the extracellular matrix (ECM), allowing them to access both the brain parenchyma and perivascular spaces. Their highly migratory nature enables them to evade complete removal during surgery, with the result that tumor recurrence is frequently observed in close proximity to the original site [5,6]. The invasive behavior is closely linked to the organization and directionality of cell movement. This process involves the remodeling of the ECM and the cytoskeleton, interactions through cell–cell and cell–ECM adhesion, and the expression of epithelial–mesenchymal transition (EMT) features [7,8]. Recurrence is also related to the tumor’s intrinsic and acquired chemoresistance mechanisms, including the expression of DNA repair enzymes (e.g., O-6-methylguanine-DNA methyltransferase, MGMT), altered drug efflux systems, metabolic reprogramming, and the survival of therapy-resistant glioblastoma stem cells (GSCs), which play a key role in tumor growth and in therapeutic resistance, exhibiting low responsiveness to both chemotherapies and radiotherapies, which further decreases following repeated chemoradiation treatments [4,9,10,11]. Another major challenge for GB treatment is its extreme cellular and molecular heterogeneity, which includes genetic mutations, different epigenetic profiles and complex interactions with the tumor microenvironment (TME). This heterogeneity makes it extremely difficult not only to treat effectively multiple patients but also to target all tumor cells of the same patient at a given time within different regions or over time as the tumor evolves [3,12,13,14]. Therapeutic development is further hindered by the presence of the BBB, which is a highly specialized and selective interface that preserves the central nervous system homeostasis by shielding the brain from detrimental substances circulating in the bloodstream, including pathogens like viruses. Its complex architecture consists of several layers, primarily composed of non-fenestrated endothelial cells of the brain microvasculature, tightly connected by junctional complexes. The BBB’s protective function arises from a combination of physical, transport-related, and metabolic mechanisms within the endothelial cells, which are finely regulated through interactions with various vascular, immune, and neural cell populations [15,16]. While this barrier is crucial for maintaining brain health and function, in pathological conditions, like GB, it poses a major obstacle to the effective delivery of therapeutic agents, thereby reducing treatment efficacy [16,17,18,19]. Even though the integrity of the BBB is significantly impaired in GB, this is not beneficial for therapeutic drug delivery. Instead, the leakage of the BBB, also named the blood–tumor barrier (BTB), which has unique features with respect to the BBB, allows infiltration of tumor cells, inflammatory mediators and pro-tumor signals, contributing towards the GB’s highly invasive nature [20,21,22]. Due to all these features, clinical management of GB patients is nowadays extremely challenging. Figure 1 summarizes the main characteristics of GB and the principal therapeutic treatments currently used, which, however, are still inefficient in avoiding tumor recurrence.

Emerging therapeutic strategies involve immunotherapies, nanoparticles and targeted therapy [3,23]. Immunotherapy proposes to use checkpoint inhibitors, vaccines or chimeric antigen receptor T (CAR-T) to promote an immune response against cancer cells [21,24,25]. Nanoparticles include extracellular vesicles naturally produced by cells or biomimetic nanostructures, combining the advantages of both artificial and natural nanocarriers. Both seem promising candidates for drug delivery to counteract GB progression and invasion [4,26,27]. Targeted therapy is designed to selectively inhibit molecular pathways dysregulated in GB cells without impairing normal cells, as for example Epidermal Growth Factor Receptor (EGFR), Platelet-Derived Growth Factor Receptor (PDGFR), Vascular Endothelial Growth Factor (VEGF) or Phosphatidylinositol 3-Kinase (PI3K)/Protein Kinase B (AKT)/Mechanistic Target of Rapamycin (mTOR), all critical for tumor progression [28,29].

However, despite considerable efforts to elucidate GB tumorigenesis pathways and develop novel treatments, many therapies that show promise in preclinical studies do not translate into effective patient outcomes. Differences observed between preclinical and clinical outcomes underscore the importance of developing a robust translational platform capable of faithfully mimicking the biological, molecular and structural aspects of GB (including its heterogeneity, invasiveness, and interactions within the brain microenvironment). An international panel of clinicians and researchers convened by Cancer Research UK identified, already in 2019, the development of more accurate and predictive preclinical models as one of the main challenges to face for curing patients with brain tumors [30]. This is the context in which our review is located, providing a comprehensive and updated overview of the current models available in GB research. The review aims to highlight specific advantages, limitations and applicability of the different models in the context of translational oncology. Our final goal is to provide awareness of the current strategies for GB biological and therapeutic assays, as well as to suggest future directions towards a multimodal integrated approach to GB preclinical research.

## 2. Glioblastoma Preclinical Models: An Overview

**Figure 2:**
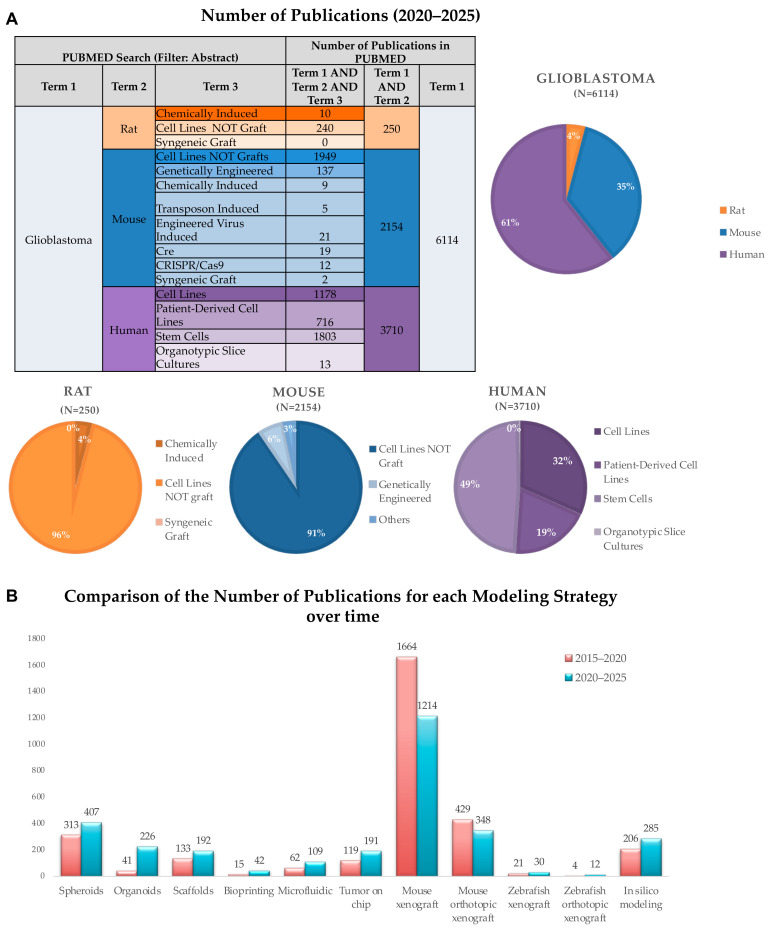
Trends in Glioblastoma modeling based on publication data. (**A**) PubMed analysis of articles published over the past five years on different glioblastoma (GB) model systems. The search strategy included (i) Term 1 (“Glioblastoma”) only; (ii) Term 1 AND one Term 2 (rat, mouse, or human); and (iii) Term 1 AND one Term 2 AND one Term 3 (referring to specific modeling approaches). Searches were limited to the article abstract field and to the period from August 2020 to July 2025. The table presents the total number of publications on GB, broken down by species and model type. The accompanying pie charts illustrate the distribution of GB modeling across species, as well as the relative proportion of different modeling strategies within each species. (**B**) Comparison of the number of publications referring to different GB modeling strategies between two time periods: August 2015–July 2020 and August 2020–July 2025. Modeling strategies include 3D culture systems (spheroids, organoids, scaffolds), bioengineering platforms (bioprinting, microfluidics, tumor-on-chip), animal models (xenografts and orthotopic xenografts), and in silico approaches. Searches were performed using the term “Glioblastoma” in combination with the term identifying each modeling strategy, filtering for article abstracts within the defined time intervals.

Preclinical models play a key role in elucidating GB pathology and testing the efficacy, dosage and safety features of therapeutic factors prior to entering clinical phase trials. To achieve an ideal experimental setup, GB preclinical models should satisfy the following specific standards: (i) their genetic characteristics and intratumor heterogeneity should resemble those of patients with GB; (ii) they should effectively replicate the GB microenvironment and its relationship with the human brain; and (iii) they should be reproducible and consistent over time [31]. Although the first GB models were unable to meet all these requirements, they enabled scientists to begin understanding and analyzing the characteristics of GB and to start testing potential therapeutic treatments. Today, increasingly sophisticated and complex GB models are being developed that closer and closer resemble human GB tumors. In the following paragraphs, GB models are first divided according to whether they originate from animals or humans, and then according to the different modeling strategies in the absence or presence of the TME cells. The specific strengths, weaknesses, purposes and examples of applications have been discussed. Figure 2A offers a panoramic view of how the different GB model systems have been recently represented in the literature, by showing the number of articles that mention each specific one in the abstract, broken down by the August 2020–July 2025 time period.

The table shows the total number of publications on GB (Term 1) per species (Term 2) and model type (Term 3), and the pie charts summarize the relative distribution of each model type within each species. The majority of GB studies of the last five years utilized human-based models (61%), followed by mouse (35%) and rat models (4%). Rodent models were predominantly based on cell lines not grafted (96% rat and 91% mouse), whereas human models showed greater diversity, including stem cell-derived models (49%) and patient-derived cell lines (19%). Figure 2B compares the number of articles published in two different time periods. (August 2015–July 2020 and August 2020–July 2025) on the different GB modeling strategies, including 3D culture systems (spheroids, organoids, scaffolds), advanced bioengineering approaches (bioprinting, microfluidics, tumor-on-chip), animal models (mouse and zebrafish xenografts), and in silico modeling. Notably, there is an overall increase in the use of 3D modeling (spheroids, scaffolds, and especially organoids) and in silico approaches in the 2020–2025 period, with a relative decline in mouse xenograft studies.

## 3. Glioblastoma Origin

### 3.1. Animal

**Figure 3:**
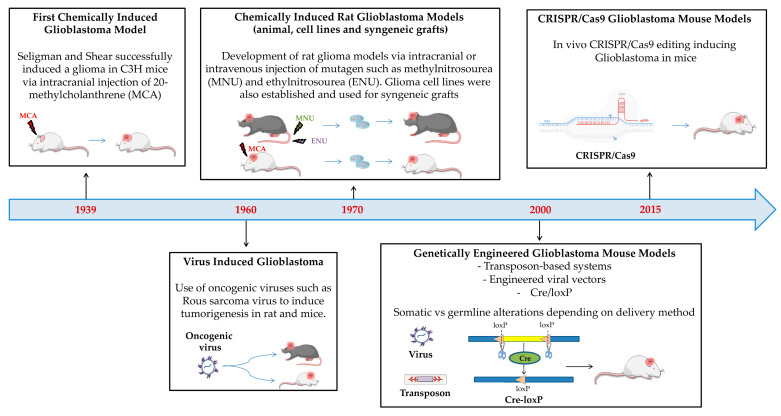
Timeline emphasizing the principal milestones in the development of GB models derived from animal sources.

Here, we provide an overview of the main GB models derived from animal sources, showcasing their origin, key features, strengths, weaknesses and purpose, as also summarized in Table 1. The development over time of the GB models of animal origin is reported in Figure 3.

#### 3.1.1. Origin and Characteristics

##### Chemically and Genetically Induced In Vivo Models

For several human pathologies, including cancer, research has attempted to establish animal models that could resemble the pathological features of human diseases. According to a general consensus, valid GB animal models should possess the following requisites: (1) origin from glial cells; (2) predictable and reproducible tumor growth rate; (3) glioma-like growth characteristics within the brain such as BBB leakage, an invasive behavior and neovascularization; (4) sufficient host survival time for in vivo analysis and therapeutic studies; (5) possibility to propagate and clone them in vitro as continuous cell lines and in vivo by serial transplantation [32]. Different GB models have been first developed in rodents by directly inducing tumor formation through mutagen injections intracranially or intravenously. In 1939, the first glioma tumor was successfully induced by Seligman and Shear after implanting the mutagen 20-methylcholanthrene intracranially into C3H mice [33]. Since the 1960s, viruses, such as Rous sarcoma virus, have also been used to induce tumorigenesis in both rats and mice [34,35], even with several limitations, such as the incomplete penetrance of the induced tumor, safety concerns and extremely higher costs [36]. Since the late 1960s, several rat GB models (i.e., C6, 9L, T9, RG2, F98, CNS-1, BT4C and RT-2) have been produced by specific mutagen injection and used to study GB biology as well as for evaluating the efficacy of various therapeutic strategies [32,37]. Among the most studied, C6 glioma rat models were produced by repetitively administering methylnitrosourea (MNU) over a period of approximately 8 months to adult outbred Wistar rats, whereas the 9L gliosarcoma tumor model was obtained by injecting MNU for 26 weeks into inbred Fisher rats [38,39]. RG2 and F98 gliomas were both chemically induced by a single ethylnitrosourea (ENU) injection to pregnant Fischer rats, the progeny of which developed brain tumors that subsequently were propagated in vitro and cloned [40]. Until the 2000s, rat tumor models were more widely used than mouse ones, which were mainly limited to the GL261 mouse models, obtained by intracerebral injection of methylcholanthrene into C57Bl/6 mice, followed by serial intracranial and subcutaneous transplantation of tumor fragments [41,42]. C6 and 9L rat glioma models had a circumscribed growth pattern, whereas both rat F98 and GL261 developed infiltrative tumors [37]. With the incoming possibility of genetically manipulated mouse embryonic stem cells, transgenic cancer mouse models definitely gained the upper hand. Genetically engineered mouse models (GEMMs) can be produced by different methods, including Cre/loxP traditional or inducible systems, transposon usage, CRISPR/Cas9 systems and engineered viruses (both retrovirus and lentivirus) [36,37,43]. The turnaround time is extremely variable, from 10 to 14 months for the Cre/loxP system to a few months for the CRISPR/Cas9 strategy, and a few weeks in the case of transgene delivery through engineered viral vectors. The genetic alteration for most systems can be in the germline or somatic; in the case of direct virus injection, it is only somatic [36,43].

##### Cell Lines

In most cases, cells were derived from the GB animal models obtained, propagated in vitro, and cloned to obtain stable cell lines. Cell lines were named after the glioma tumor model from which they were derived, and used in both in vitro testing and in vivo transplants. The most used rat cell lines were the C6, 9L and F98, each one carrying specific gene mutations, some of them in common with the ones found in human GB [32,37]. C6 cell line, for example, shows higher expression of Ras oncogene with consequent Ras pathway activation [44], whereas 9L cells carry mutation on p53 tumor suppressor gene and show higher EGFR expression [45,46], and F98, overexpressing Ras, Platelet-Derived Growth Factor B (PDGF-B), cyclin D1, cyclin D2, and EGFR and downregulating Breast Cancer gene 1 (BRCA1) is the most similar to human GB [46,47]. Among the most used mouse cell lines, there were GL261, sharing many genetic mutations with human GB, including Ras point mutations and activation, along with loss of oncosuppressor genes such as p53 [48]. Even though chemical-induced animal GB models have long been outdated, several mouse and rat cell lines established from those tumors have been used for in vitro assays and allograft models [36] as well as for the setup of GB 3D culture models.

##### Syngeneic Grafting Models

The grafting of tumor cells inside an animal model ensures a steady and constant supply of nutrients, growth factors and oxygen, which is ideal for the study of tumor progression. Syngeneic grafts, allografts and xenografts can be distinguished, depending on whether the donor and the host have the same genetic background (syngeneic), have different genetic backgrounds but are of the same species (allografts) or belong to different species (xenografts). Moreover, transplants can be heterotopic, if they occur in a different location with respect to the site of origin, mainly subcutaneously, or orthotopic, when engrafted cells are implanted in the same location, which, for GB, is the brain [49]. Syngeneic orthotopic GB grafts were first used in rats of the same strains to study GB progression and response to therapy, avoiding immune rejection [32,36]. Among the most widely studied, there was the 9L rat gliosarcoma model, characterized by a high immunogenicity and a circumscribed tumor growth pattern, with sharp margins and poor invasion into the contiguous normal brain, and F98, showing exactly the opposite characteristics [37]. For this reason, F98 is definitely more similar to human GB, characterized by high invasiveness. For C6 instead, having been generated in outbred Wister rats, there is no syngeneic host in which it can be propagated, but allografts in Wister rats have been performed. C6 cells are highly immunogenic and poorly invasive [50]. The mouse cell lines used in the allograft mouse model include CT-2A and 4C8, but especially GL261, which more closely resemble human GB features and are characterized by high immunogenicity [36].

#### 3.1.2. Purposes, Strengths and Weaknesses

The use of animal in vivo models has the strong advantage of allowing for the study of the interaction between the developing tumor and the TME, as well as the behavior of the BBB or BTB. These aspects are particularly relevant with respect to both GB pathogenesis and therapeutic development. Rat and mouse brain tumor models have been the most widely used in experimental neuro-oncology, and this has led to the development of several approaches for the treatment of human GB. Rats were more widely used than mice until the 2000s, despite higher costs of purchase and maintenance. In fact, they had undoubted advantages, such as the larger size of their brains (1200 mg vs. 400 mg), facilitating intracranial stereotactic procedures, and consequently larger tumor sizes and longer time until death, which allowed for better in vivo imaging and prolonged testing of therapeutic agents [32]. However, with the onset of genetic manipulation, which is much easier in mice than in rats, transgenic mouse models prevailed. Independent of the strategy used, transgenic mice were based on the introduction of a specific mutation in the target gene(s). Therefore, differently from the tumor models generated by a general and untargeted mutagenesis, transgenics have a precisely known genetic signature. This feature has the advantage of allowing for the study of the effect of specific targeted genetic mutations, but, at the same time, it cannot reflect the typical GB heterogeneity. Syngeneic transplant models in both mice and rats have been used to study the immune mechanism for radiation therapy, immune checkpoint therapy, VEGF therapy and vaccine therapy [36]. Rodent tumor models have been mainly used in neuro-oncology to evaluate the efficacy of a plethora of therapeutic treatments, including chemotherapy, radiation therapy, antiangiogenic therapy, photodynamic therapy, oncolytic viral therapy, gene therapy and treatment with proteosome inhibitors or toxins [36,37,47,51,52,53,54,55,56,57]. Within a hypothetical preclinical pipeline, in vivo models should ideally be employed in the later stages, once therapeutic candidates have been identified, validated in simpler and more targeted models, and their mechanisms of action have been at least partially elucidated. The typically long time required for model development and analysis, the need for access to an animal facility, medium-to-high housing costs, inherently low throughput, as summarized in Table 1, and, last but not least, ethical concerns are critical factors to consider. Finally, the strong limitation of both chemically induced, genetically engineered and syngeneic grafting rodent models, despite a similar genome, relies on the genetic and phenotypic difference between rodent and human tumorigenesis processes. Tumor growth and progression might use different paths and, therefore, targeting molecules and therapeutics having a positive antitumoral outcome in animal models might fail to function in patients [58]. In addition, even though rodent and human brain architectures have several similarities, the human brain and neocortex are significantly more complex, and human glia possess different features from those of mice [59,60,61]. For these reasons, deriving and studying human GB cells from patients to compare the different results obtained is fundamental.

### 3.2. Human

Here, we describe the main GB models originating from human sources, including their origin, main attributes, benefits, constraints and purpose. Table 2 provides a summary of the more relevant info, highlighting the needed effort in terms of cost and time, model representativity with respect to patient GB, model complexity, throughput and common applications.

**Figure 4:**
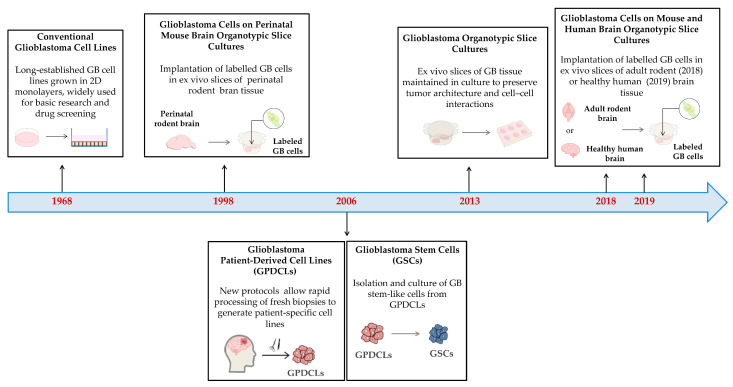
Timeline showcasing the key milestones in the development of glioblastoma (GB) models derived from human sources.

Figure 4 summarizes the key milestones in the development of GB models from human sources.

**Table 2:** Main glioblastoma models of human origin.

Origin	Model Type	Generation	Effort		Representativity Respect to Patient GB	Complexity			Purpose	Throughput
			Cost	Time		ECM	TME	BBB	Applications	
**Human**	*Conventional cell lines*	Isolation and immortalization	L	L/M	L Differ from patient GB both genetically and phenotypically; Can accumulate genetic mutations in culture	-	-	-	Cell proliferation, metabolism, viability, and migration; Drug assays; Resistance to therapy	H
	*Patient derived cell lines*	Isolation from fresh biopsies and limited passage in culture	M	L/M	H Maintain the genotypic and phenotypic features of the tumor of origin; Reflect GB patient variability	-	-	-	Cell proliferation, viability, and migration; Response to therapy; Personalized medicine	M
	*Glioblastoma stem cells*	Isolation from fresh GB biopsies or iPSC reprogramming or conventional cell lines derivation; culturing in absence of serum and presence of specific factors (EGF, bFGF)	M	L/M	H if derived by GB patients; Can differentiate also in TME cells	-	-/+	-	Cell proliferation and invasion; Resistance to therapy; In vivo tumor formation; Personalized medicine (if derived by GB patients)	M
	*Patient derived organotypic slice culture*	Removal of GB tissues, cutting in sections and culturing for limited time	M/H	M/H	H Maintain the genotypic and phenotypic features of the tumor of origin; Reflect GB patient variability	+	+	+	Cell proliferation, death, and invasion; immune response; drug assays; resistance to therapy; personalized medicine	L

Abbreviations: BBB, blood–brain barrier; bFGF, basic fibroblast growth factor; ECM, extracellular matrix; EGF, epidermal growth factor; GB, Glioblastoma, H, high; iPSC, induced pluripotent stem cell; L, low; M, medium; TME, tumor microenvironment.

#### 3.2.1. Origin and Characteristics

##### Conventional Cell Lines

In 1968, glioma human cell lines were first isolated to obtain immortalized and stabilized long-term cultures [62]. During the following decades, several cell lines (i.e., U87-MG, U251-MG, T98G, LN229, GBM) were established from GB patients and immortalized to make them proliferate indefinitely. Despite several common features, the different cell lines show high genotypic and phenotypic heterogeneity, largely due to the heterogeneity of the tumors of origin as well as of the different cell types inside the same tumor [63]. Human tumor cell lines have been traditionally used and are still in use to understand the molecular basis of human GB and to test candidate drugs affecting specific GB features [64,65,66,67,68]. Among GB cell lines, the most widely studied are the U87-MG, which was isolated from a malignant glioma from a male patient and characterized by an epithelial morphology. U87-MG has been studied in a plethora of in vitro functional assays to test cell viability, proliferation, migration and the chemoresistance of GB cells. Moreover, it has been successfully transplanted subcutaneously and intracranially in mouse models, where it show fast growth, with a median survival of around 22 days [69]. U251-MG is a pleomorphic/astrocytoid cell line also used for both in vitro assays and mouse xenografts, where they grow and form tumors very fast, with a median survival of around 28,5 days [69]. The various cell lines differ in their gene expression profiles, and, therefore, in their functional characteristics, including proliferation and migration rate, invasion and colony formation capacities [63,70].

##### Patient-Derived Cell Lines

Cell lines derived from patient GB were first established in 2006 by Lee and coworkers [71]. GB patient-derived cell lines (GPDCLs) differ from traditional cell lines because they are derived from fresh biopsies processed within 2–3 h using specific protocols that allow for the preservation of the in vivo biology of the tumor of origin. Therefore, GPDCLs have a better chance of recapitulating the GB genetic profile, gene expression pattern and its histological features [72,73,74]. Interestingly, since 2015, at least two biobanks have been developed for collecting annotated and validated GPDCLs, classified according the transcriptional GB subtypes, mesenchymal, proneural, neural and classical [75,76]. These collections are open resources that undoubtedly constitute a precious opportunity for both basic and translational GB research. The culture conditions used for deriving GPDCLs also preserve GSC identity and features [69].

##### Glioblastoma Stem Cells

GSCs, due to activated signaling pathways, such as Wnt/β-catenin, Notch, PI3K and Janus kinase)/signal transducer and activator of transcription (JAK/STAT), are principally responsible for resistance to radiation and conventional chemotherapeutics, as well as for the invasive GB behavior, and, last but not least, for tumor relapse. These properties render GSCs particularly relevant for GB therapy. Moreover, GSCs are capable of differentiating into non-tumorigenic cells, such as endothelial cells, that are part of the tumor microenvironment [77]. Therefore, recent studies specifically aimed to enrich GSCs in immortalized or patient-derived cell lines (PDCLs), by both two-dimensional (2D) as well as three-dimensional (3D) culture [78,79,80,81]. Other approaches for obtaining GSCs are based on the reprogramming of human-induced pluripotent stem cells (hiPSCs) to neural progenitor cells that can acquire the characteristics of GSCs, including the ability to form GB tumors when orthotopically transplanted into mouse brains [82,83].

##### Patient-Derived Organotypic Slice Cultures

The organotypic slice cultures originate from GB tissues acquired during neurosurgical procedures, cut into thick slices (hundreds of μm in diameter), and maintained on membranes in 6-well plates for up to four weeks, maintaining their cellular structure. This approach was set up in 2013 by Metz and coworkers, and was first used to test the therapeutic effect of TMZ, X-ray or carbon-ion radiation therapy, monitoring cell proliferation, cell death and DNA double-strand breaks [84]. The study revealed the suitability of the slices as an experimental model to dissect mechanisms of therapy resistance, as well as an assay platform for novel personalized treatments. An alternative approach to studying GB cell invasion is to derive organotypic slices from healthy mouse or rat brain tissue and study the migration behavior of previously labeled GB tumor cells (single cells or tumor spheroids) that have been implanted. This approach was first used in 1998 on slices from perinatal rat brain [85], and then optimized from adult mouse brains, which better recapitulate features of the adult GB tumor microenvironment [86]. In 2019, slices were obtained from human healthy tissues surrounding the excised GB. Implantation of GB patient-derived cells (GPDCs) to the healthy brain tissues of the same patients may represent a powerful model to study invasive GB behavior as well as to develop novel personalized treatments [87,88].

#### 3.2.2. Purpose, Strengths and Weaknesses

Human immortalized cell lines constitute a very simple and well-characterized system that can be easily cultured and studied by using the most widely diffused proliferation, migration and invasion assays. Cell line cultures are commercially available, not particular expensive and require only basic cell biology expertise. Moreover, they do not pose any ethical concern related to the use of animal models or human biopsies. Cell lines are also suitable for the use in high-throughput screening, for an initial evaluation of huge amounts of compounds with possible antitumoral activities. Therefore, in vitro assays with GB human cell lines are still valuable complementary options, providing a simple and easy-to-obtain basis for further ex vivo and in vivo research. However, cell lines are not able to resemble the heterogeneity and complexity of GB tumors and their intricate relationships with TME (Table 2). Most cell lines were established decades ago and may have lost key features respect to the tumors they were derived from. Moreover, traditional 2D models have very limited ability to simulate the in vivo tumor conditions. To have more reliable results, it is advisable to utilize at least multiple cell lines with different genetic backgrounds. The origin of the cell line, the number of passages in culture and the absence of Mycoplasma are also key parameter to check to improve reliability and reproducibility of the results. Finally, 3D culture models might also improve the reliability of the cell culture system [69,89].

Primary cells and tissues, which are derived directly from patients, possess invaluable features that render them extremely precious for preclinical studies. Maintaining biological relevance, GPDCLs and GB organotypic slice cultures closely reflect the tumor of origin, and the complex architecture of in vivo conditions. Moreover, thanks to their donor-specific variability, primary cells and tissues allow researchers to explore the impact of genotypic and phenotypic diversity on tumor cell behavior, and therapy response, opening the way to the development of personalized treatments [90].

GPDCLs are an important resource for the development of clinically relevant ex vivo models, since they are both genotypically and phenotypically closer to the original tumors they were derived from. However, GPDCLs are difficult to establish and maintain in tissue culture, each time requiring specific experimental procedures that closely depend on the patient and tumor [84]. Culturing methods, 2D or 3D, also influence genetic stability, with 3D cultures being more efficient in preventing genetic changes compared to the tumor of origin [91]. Moreover, these cell lines require a very long time to form in vivo tumors in xenograft animal models, varying from 2 to 11 months [69,89]. Therefore, difficulties in obtainment, time constraints and inconsistent culture limit their use. GSCs, on the other hand, are very interesting systems that bring together the main GB features related to a poor prognosis (chemoresistance, invasiveness, relapse), and, therefore, are a suitable model for therapeutic investigation. However, culture conditions might strongly affect GSC homogeneity and propagation and, even more importantly, may cause the loss of specific mutations, including the EGFR ones, which are present in almost 50% of the GB biopsies, weakening consequent analysis [77].

The organotypic slice cultures include several advantages, such as: (i) the presence of the organotypic matrix and TME components; (ii) open access allowing treatment and direct observation over extended periods of time; and (iii) collection of supernatants for analysis. On the other side, slice cultures are costly and time consuming, and, due to their intrinsic nature and derivation, individually unique and less suitable for well-controlled reference experimental systems [69,89]. Both GPDCLs and organotypic slices were used to study GB cell proliferation, migration, invasion, and susceptibility to treatments. Both models could be useful at an intermediate/advanced stage of a hypothetical preclinical pipeline, once evidence of efficacy and safety has been obtained using simpler models. They might be extremely relevant for studying patient variability and different susceptibility to treatments, and for linking the anti-tumor response to a particular genotype, with a view to developing personalized medicine protocols. Moreover, implantation of labeled GPDCs on brain organotypic slice culture derived from the healthy tissues of the same patient can offer the unparalleled opportunity to study the interaction among tumor cells and the brain microenvironment, comprising both glia, vascular and immune cells, and to monitor personalized responses to different therapies.

## 4. Cell Culture Modeling Strategies

### 4.1. 2D Models

#### 4.1.1. Origin and Characteristics

The 2D monolayer cell culture is the conventional culture system of cells growing in adhesion in culture medium containing serum and all the nutrients necessary for cells to grow and proliferate.

#### 4.1.2. Purpose, Strengths and Limitations

A 2D cell culture is an easier and cost-effective way of culturing, for which standardized protocols and commercially available reagents and tests are available. It is characterized by high homogeneity of cell population, in which all cells have the same access to oxygen and nutrients [90]. Two-dimensional models have been used in a plethora of assay, including GB cell proliferation, toxicity and migration assays and have greatly contributed to drug discovery and understanding of GB. However, they have two great biases that 3D culture systems avoid. First of all, 2D culture models are subject to both genotypic and phenotypic drift, after being subjected to repeated passaging in culture for a considerable amount. This is true for commercial cell lines, but also for patient-derived ones. Second, they fail to represent the complexity of the cell-to-cell interactions which characterize the tumor and its microenvironment. These limitations have strongly restricted the usage of 2D culturing systems over time, especially in immortalized cell lines, to preliminary and/or high-throughput assays. In the meantime, more suitable and complex models have been developed, and their usage in preclinical assays is increasing [43,49,77], as is also shown in Figure 2B.

### 4.2. 3D Models

#### 4.2.1. Origin and Characteristics

**Figure 5:**
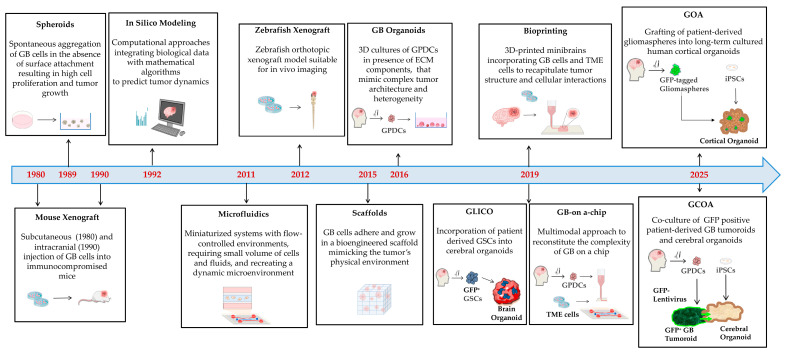
Timeline showcasing the key milestones in the development of GB modeling strategies. Abbreviations: ECM, Extracellular Matrix; GB, Glioblastoma; GCO, Glioblastoma Cortical Organoid; GCOA, Glioblastoma-Cerebral Organoid Assembloid; GFP, Green Fluorescent Protein; GLICO, Cerebral Organoid Glioma; GPDCs, Glioblastoma Patient-Derived Cells; GSCs; Glioblastoma Stem Cells; iPSCs, induced Pluripotent Stem Cells; TME, Tumor Microenvironment.

Recently, various types of GB 3D models have been developed to culture established cell lines, primary tumor cells as well as tumor biopsies. All 3D culture models are characterized by complex interactions among tumor cells, which aggregate then resemble the tumor growth inside the organism. However, they differ based on the presence or absence of ECM components or external scaffolds, which influences and guides tumor cell aggregation and growth. Here, we report the different 3D model systems actually available, in order of increasing complexity and concomitant similarity to in vivo tumorigenesis. The key milestones in the development of GB modeling strategies are shown in Figure 5, whereas specific highlights, pitfalls and benefits, as well as applications, are outlined in Table 3.

##### Spheroids

Tumor spheroids form by the spontaneous aggregation of tumor cells, in the absence of any surface attachment, and even ECM components, thus resulting in high levels of cell proliferation and tumor growth [43,77]. Following intercellular contact, tumor cells produce adhesion molecules, such as cadherins and integrins, on the cell surface, which promote cell survival and allow for the growth of spheroids as a compact structure [92,93]. Tumor spheroids were first generated in the early 1970s by Sutherland and coworkers [94], and, since then, a plethora of spheroid methods have been developed. Three different spheroid models can be identified, based on the cellular sources and methodology used. Multicellular tumor spheroids (MCTSs) were the first GB spheroid model to be obtained, in 1989 [95]. They are typically established from cancer cell lines in conventional media supplemented with FBS, and, differently from conventional 2D models, are grown as spheres in a suspension culture or under other conditions that promote cell–cell adhesion [95]. MCTS are clonal, easy to culture and genetically manipulate and, even though they show little histological resemblance to the primary cancer, are still able to mimic its metabolic, proliferative and chemoresistance properties [79,96]; for these reasons, they are used in high-throughput drug screening [97]. On the other side, GB-derived spheroids or gliomaspheres have been generated by the mechanical or enzymatic dissociation of tumor specimens into a single cell suspension followed by growth in absence of serum and in the presence of supplements, such as B27 and N2 nutrient mix, epidermal growth factor (EGF) and/or basic fibroblast growth factor (bFGF), to positively select for GSCs [78,80]. Finally, organotypic multicellular spheroids (OMSs) are established after extremely gentle mechanical or enzymatic dissociation of cancer tissues in such a way as to also save surrounding non-tumor cells, such as stromal, vascular and immune components of the TME. As a result, OMSs, differently from the other two types, are similar to ex vivo explants, generally retaining many histological features and the cellular heterogeneity of the primary GB, and faithfully reproducing the TME [78,80].

##### Tumor-like Organoids or Tumoroids

Organoids are complex structures that aim to recapitulate the whole organ starting from stem cells (embryonic or induced pluripotent stem cells) that are left to develop and differentiate in 3D culture systems [89,98]. In 2013, neural organoids, or minibrains, were generated from hiPSCs [99]. Tumoroids are a particular type of organoids that are derived from patient cancer tissues. GB cells self-assemble and interact with each other to form 3D tissue-like structures, following specific protocols, usually involving Matrigel or other components of the ECM [43,77]. The presence of ECM components results in a higher level of structural complexity of the organoids, with respect to spheroids, and the acquisition of an architecture that is closer to that of the tumor of origin [93]. The first GB organoids (GBOs) were generated in 2016 by adapting the methodology previously used for mini-brains [100]. Successfully implanted into mouse brains, these organoids produced more sophisticated and representative models than basic GPDCLs. A biobank of patient-derived GBOs, each one of which showed parental tumor heterogeneity, was established as a precious resource for personalized GB therapy [101]. However, derivation of organoids from patient tumors was challenging in terms of both cost and time. An alternative strategy has also been developed based on the induction of GB into brain organoids through genetic engineering by the CRISPR/Cas9 system [102,103]. Recently, technological progress has also enabled the development of more precise, customizable and physiologically relevant GBOs that also show TME components [104]. Novel, faster systems have been established to generate and bank patient-derived GBOs directly from fresh tumor tissues, without the need for the separation of single cells [105,106]. This approach is extremely significant, in fact it was used in a unique trial design for the real-time assessment of immunotherapy in patients with recurrent GB [107].

##### Cerebral Organoid Glioma (GLICO) and Other Co-Culture Systems

In 2019 a brilliant system was set-up by Linkous and coworkers, based on the co-culture of GSCs derived from patients into human cerebral organoids obtained by human embryonic stem cells or hiPSCs [108]. Patient-derived GSCs, labelled with GFP, were successfully incorporated, after one week, into the miniature brains, and they formed infiltrative tumors with a pattern strongly resembling the one commonly found in surgical GB specimens. The obtained Cerebral Organoid Glioma (GLICO) reflects the same genetic mutations and activated signaling pathway of the tumor of origin [108]. The GLICO model was found to have the highest correlation with the primary patient tumor with respect to the other three GSC-derived models, which are gliomaspheres, GBOs and patient-derived xenografts (PDXs) [109]. This model, therefore, represents a valuable opportunity to study GB progression, cell communication and infiltration into the brain tissues, as well as to test potential therapeutics [110]. With a similar approach, other types of coculture systems have been recently developed to recapitulate communication between GB cells and TME cells. Mangena and coworkers (2024) successfully incorporated GB primary cells (gliomaspheres) into long-term cultured human cortical organoids, containing the major neuroglial cell types found in the cerebral cortex. The resulting Glioblastoma cortical organoids (GCOs) faithfully recapitulate tumor heterogeneity and intercellular communication in GB patients [111]. Moreover, Kim and coauthors (2025) co-cultured GFP-positive patient-derived GB tumoroids and human cerebral organoids and established a Glioblastoma-cerebral organoid assembloid (GCOA) model, suitable for the study of the process of the invasion of tumor cells into the surrounding microenvironment [112]. Conversely, Zhou and coworkers (2024) set-up a co-culture of mCherry-labeled GBOs with GFP-labeled neurons and verified its application in cancer neuroscience to investigate communication between GBs and neurons [105].

##### Scaffold-Based Models

Differently from previous ones, scaffold-based models take advantage of biomimetic ECM-like materials, which can support and structure 3D cell culture, miming the interaction of cancer cells with each other and the surrounding ECM [43,90]. Therefore, GB cells do not freely assemble, as in GBOs, but adhere and then grow and maintain following the scaffold structure into which they are embedded. In 2015, Heffernan and coauthors developed a bioengineered 3D scaffold, a hydrogel obtained by cross-linking of hyaluronic acid and gelatin, to study GB proliferation and invasion [113]. Since then, a great effort has been produced to develop more and more suitable cell supporting materials that are able to closely resemble physiological ECM in terms of pore size and porosity. Moreover, scaffolds with post-manufacturing tunability, reproducing the ECM continuous remodeling abilities, have been also proposed [77]. Supports can be divided, based on the type of polymer, into natural- (i.e., bio-polyamide, bio-polyethylene, protein- or polysaccharide-based) or synthetic-derived (i.e., polyamide, polystyrene, polyethylene, polycaprolactone, polyanhydrides), and, depending on the rigidity of the material, into solid and hydrogels [77,90]. Synthetic scaffolds, however, can have less biocompatibility than natural scaffolds, and might require surface modification for cells to attach and grow on them. A very interesting type of scaffold has been recently developed, based on decellularized tissues, in which cells were removed, leaving only natural ECM components [90]. Co-culture with non-tumoral cells is also possible and can mimic interaction within the TME [90].

#### 4.2.2. Purpose, Strengths and Limitations

Two-dimensional cell cultures are not able to mimic the in vivo spatial complexity of the in vivo environments, as in 3D system models. Tumor cell adhesion to form 3D aggregates resembles in vivo tumor growth, with the same variability in the access to oxygen, nutrients, metabolites and growth factors, and often the acquisition of different polarity and phenotypes from the various tumor cells inside the same tumor aggregate. GB spheroids are the simplest and most popular 3D cell cultures. They show a multilayered structure with an inner layer of hypoxic and necrotic cells, an outer layer of actively proliferating cells and a middle layer of quiescent cells. This structure resembles in vivo tumor structure and recapitulated tumor properties, including response to therapy. For these reasons, GB spheroids have been largely utilized in drug screening assays [93].

Organoids are characterized by higher cell density, more physiological intercellular interactions and a more complex spatial organization than spheroids. Moreover, due to its formation process in absence of a support, GBO culture offer major versatility and customizability in terms of culture protocols and experimental design, but on the other side, show high heterogeneity of cell composition and structure and lower control and reproducibility than scaffold-based models [4,90]. Recent technological developments, however, further refine GBO modeling by starting directly with fresh biopsies, reducing the time and cost of generation and preserving the cytoarchitecture, cell–cell interactions and TME components of the parental GB. Interestingly, this approach has also been used for the real-time evaluation of ongoing therapy in patients with recurrent GB [105]. GLICO and other coculture systems (GCO and GCOA) inherit the same disadvantages as organoids, with cost and time to culture being some of the major challenges to face. However, they provide a precious method for studying GB biology in a human brain microenvironment, in particular GB growth and infiltration into brain tissues and response to therapy.

Finally, the use of a scaffold on which tumor cells adhere and grow allows for the development of controlled and reproducible tumor models. Moreover, bioactive signals or candidate drugs can be incorporated into scaffolds to create a concentration gradient and allow for the controlled release to guide tumor formation or to study drug response, respectively [90]. OMS and direct explant-derived GBOs are the ones that, by origin, contain non-tumoral cells of the TME. More sophisticated models, such as GLICO, GCO and GCOA, have been developed, by co-culturing tumor cells with stromal, immune, endothelial or neuronal cells, to get closer to in vivo physiological GB. In the following paragraph, modeling strategies more suitable for mimicking and studying TME interactions and BBB permeability are described.

## 5. Modeling of the Interactions with Glioblastoma Microenvironment and Blood–Brain Barrier

### 5.1. In Vitro or Ex Vivo

#### 5.1.1. Origin and Characteristics

##### Bioprinting

As scaffold-based models, bioprinted models also use biomaterials in which cells are dispersed. The generation of bioprinted GB models is based on the classical steps of the 3D printing procedure. First, a programmed outline of the desired structure is required, followed by image segmentation for the successive printing of the different layers that form the planned 3D tumor structure [114,115]. There are different types of bioprinters, including inkjet printers, which are supplied with a heater; extrusion-based printers, which are supplied with a piston; and laser-assisted printers. All are able to combine the bioprinting material, different types of cells, associated biomolecules and the chosen biomaterial or bioink in the bioreactor to form a complex 3D tumor–TME structure [114]. As bioinks are commonly used derivatives of hyaluronic acid, gelatin methacrylate and collagen with or without alginate, this allows for the building of a hydrogel scaffold in which the cells grow and interact [116]. The exploitation of novel biomaterials and cutting-edge technologies for tissue engineering allows for the formation of complex 3D structures, resembling the complex in vivo tumor organization, in which tumor cells establish intricate relationships with each other, the non-tumor cells of the microenvironment and the ECM. These extremely advanced structure are able to model more complex processes involved in tumorigenesis, such as, for example, the reciprocal interaction and conditioning of tumor cells and the surrounding niche, including endothelial and immune cells, and the related phenomena of angiogenesis, neovascularization, BBB leakage and immune escape [43,115,116]. Moreover, progress in the field of biomaterial and bioengineering assures a high control over the cellular and biomaterial layers, resulting in a high level of reproducibility of the bioprinted models, making them suitable for preclinical drug sensitivity assays [117,118]. The design of the 3D bioprinted model can be based on clinical images to perfectly mimic in vivo tumors from real patients or can be planned to model specific, simplified conditions and cell interactions; for instance, between GB tumor cells and macrophages or glioma-associated stromal cells [117,118]. In 2019, Heinrich and collaborators engineered 3D-bioprinted miniature brains encapsulating the RAW264.7 mouse macrophage cell line and harboring a large cavity. After the cavity was obtained, it was filled with GL261 mouse GB cell line embedded in hydrogel bioink. The minibrains unraveled the complex relations between GB cells and macrophages, showing that GB cells were able to recruit and condition macrophages, polarizing them in GB-associated macrophages or GAMs, which, in turn, induced GB cell invasiveness. Interestingly, drugs able to inhibit the interaction between GB cells and GAMs were able to reduce tumor growth and increase chemosensitivity [117]. Moreover, by utilizing 4D bioprinting to incorporate GPDCs and ECM biomaterials, Chadwick and colleagues created dynamic GBOs that can model interactions within the TME over time [119]. This method offers a novel way to investigate the evolution of GB heterogeneity and therapy resistance as they respond to environmental changes, and, ultimately, provides a strong and scalable foundation for drug screening at high throughput [31].

##### Microfluidic

Microfluidic models are based on submillimeter-scale devices equipped with etched channels, in which extremely small volumes of cells and fluids can be combined for perfusion culturing. These miniature scales come with numerous advantages, such as requiring minimal amounts of reagents, high sensitivity and resolution, and laminar flow [69,120]. Microfluidic technology had a great impact for biological applications, especially in the cancer field, allowing the controlled placement of cells and precise delivery of factors. Devices were widely constructed through a photolithographic process with polydimethylsiloxane (PDMS), characterized by high biocompatibility, flexibility, optical transparency, imaging resolution and low cost [31,69,120]. The first GB microfluidic-based model was developed by Huang and coworkers in 2011 and was composed by three communicating compartments, a seeding chamber, a receiving chamber and bridging microchannels with the aim to evaluate the migratory ability of previously isolated GSCs [121]. After that, microfluidic devices have been evolved to resemble closer and closer the in vivo tumor. Two- or three-dimensional biomimetic hydrogels (i.e., collagen, hyaluronic acid, chondroitin sulphate proteoglycans) have also been integrated into devices to mimic in vivo ECM and TME [122,123,124,125]. Moreover, concentration gradient generator microchannels and a precision syringe pump were used to allow circulation of media of a strictly controlled composition (nutrients, growth factors, substances to test) into the device, thus mimicking vasculature function and generating a dynamic and more realistic GB microenvironment [122,123]. Another development direction aims at coculturing different cell types resembling the complexity of the TME. Cui and colleagues combined mouse GB cell lines, macrophages and endothelial cells into a microfluidic angiogenesis model with controllable and biomimetic immunosuppressive conditions to study immune–vascular and cell–matrix interaction and the mechanism leading to the failure of current anti-angiogenic therapy for GB [126]. Microfluidic technology was first set-up for commercial GB cell lines and then improved for culturing GPDCLs [127,128]. In 2018, researchers were able to expand GB primary cell cultures and grow them as spheroids, with high cell viability and a high volumetric yield, whilst maintaining GSC features [127]. Moreover, different combinations and concentrations of chemotherapy drugs could be screened to identify the optimal combination tailored to individual GB patient [128]. The results obtained were consistent with the inherent TMZ GB resistance in patients. Microfluidic devices filled with GPDCLs in culture opened the way to personalized medicine approaches. Nowadays, commercial microfluidic devices are also available, and these have been successfully utilized to evaluate the antitumoral effects of new candidate drugs [129]. Microfluidic technology was also used to maintain entire GB patient-derived tissues in culture. Olubajo and coworkers (2020) developed a device composed of two layers of glass bonded together to contain a tissue chamber and a network of microchannels for permanent tissue perfusion. The device was used on 128 GB biopsies from 33 patients and allowed tumor tissue maintenance for an average of 3 days with high cell viability and histological similarity to fresh biopsies [130]. Finally, microfluidic platforms have been also designed for the production of drug delivery and manufacturing systems to test efficacy of novel GB therapeutics [131,132].

##### GB-on-a-chip

GB-on-a chip-models aim to recreate in vitro the complexity of the in vivo tumor, by co-culturing in a sophisticated 3D environment supported by analogues of ECM, both GPDCs and non-tumor cells (macrophages, stromal, endothelial cells), in a dynamic relationship. In 2019, Yi and colleagues bioprinted a GB tumor co-culturing GPDCs, endothelial cells and decellularized ECM from brain tissue in a compartmentalized cancer-stroma concentric-ring structure sustaining a radial oxygen gradient. The bioprinted model recapitulated the structural, biochemical and biophysical properties together with the patient-specific tumor resistances to treatment [133]. On the other hand, thanks to microfluidic devices, GPDCs can be cultured in a dynamic 3D environment together with stromal, immune and endothelial cells, mimicking both the TME cellular interactions, the in vivo oxygen and nutrient gradients as well as drug diffusion and permeability. Truong and coauthors combined GPDCs in Matrigel and HUVECs in fibrin gel in a GB-on-a-chip to mimic the GB vascular niche and study the effect of endothelial cells on GSC proliferation, migration and molecular features. The model was validated as physiologically relevant thorough comparative analysis with in vivo orthotopic mouse patient-derived xenograft (PDX) model [134]. Finally, microfluidics, tissue engineering, biomaterial research, bioprinting and, more recently, biosensors for better downstream analysis have been converging to develop more and more sophisticated GB-on-a-chip models. In 2020, Ozturk and co-workers created a 3D microfluidic platform comprising of bioprinted patient-derived GB spheroids integrated within two perfused vascular channels to analyze the long-term effects of TMZ treatment. Noteworthily, this study highlighted the need for multi-model treatment strategies to understand and bypass GB resistance and recurrence [135]. Nowadays, various microfluidics-based approaches have been developed to model in vitro both BBB function and tumor interaction with the immune system, both of which are key aspect for therapeutic efficacy. Toward this goal, microfluidic devices have been generated, that culture human GB primary cells into novel brain-mimetic hydrogel biomaterials or co-culture GB spheroids from PDXs with endothelial cells, pericytes and astrocytes [136,137]. Moreover, Bayona and coauthors (2025) have developed a novel microfluidic-based chip, made of cyclic olefin polymers and copolymers, and consisting of completely interconnected three chambers, that ensure direct communications among cells in the different chambers as well as continuous renewal of oxygen and nutrients, and drug administration [138]. The device allowed the study of the tumor-immune cross talk in GB, monitoring the impact of both chemical (TMZ) and physical (ECM stiffening) factors on GB-immune dynamics [138]. All these findings demonstrate the effectiveness of GB-on-a-chip technology in modeling parental GB, even with respect to more complex features such as BBB permeability and immune interactions. This technology is therefore becoming increasingly relevant for drug testing and the definition of personalized therapeutic strategies.

#### 5.1.2. Purpose, Strengths and Limitations

Both bioprinting and microfluidic technologies allow to assemble multiple cell types to study interactions among tumor cells and the other cells of the TME. This added value, however, also has a high cost in term of time and human and economic resources. Moreover, 3D bioprinting currently has various limitations, including the need to improve printing resolution and to develop suitable printing biomaterials with both the mechanical properties necessary for printing and the physiological properties for mimicking in vivo tumor [118].

Microfluidic technology, respect to bioprinting, gives the opportunity to create multiplex physical and chemical gradients to better mimic the dynamic environment which resembles closer the physiological situation in which tumor cells continually receive oxygen, nutrients and factors. Moreover, the device transparency allows real-time visualization and high-resolution in vivo imaging. There are several advantages to the use of microfluidics, including the minimal amount of cell samples and biomaterials required, the high sensitivity and the rapid analysis time. However, microfluidics possesses some limitations linked to the material of the device, which is often PDMS, as it can absorb hydrophobic components, altering their gradient inside the device and then affecting drug response and the reliability of the results. Moreover, microfluidic devices cannot restructure the shear stress observed in GB in vivo, and, to ensure the equilibrium of cytokines in the growing cells, sub-channels linking the microwells to the main channel should be included [31,69,120]. On the other hand, one major benefit of bioprinting respect to microfluidics is the possibility of adopting a one-step production process, enabling customizable architectural designs with less labor and advanced precision. Finally, GB-on-a-chip models have the potential to improve knowledge of human cancer biology, increase the speed of drug discovery and allow for the exploration of the pharmacokinetics of GB therapeutics, as well as offering promising applications in personalized medicine. Multimodal systems, incorporating microfluidics, 3D bioprinting and biosensors, have demonstrated promise in evaluating various GB therapeutic response in a physiologically representative environment [31,139]. Advanced GB-on-a-chip models can obtain a greater resemblance to parental GB, as well as being more standardized and reproducible, within a highly customizable and cheaper platform.

### 5.2. In Vivo Graft Models

#### 5.2.1. Origin and Characteristics

Nowadays, the GB animal models used are basically generated by xenografts of different human GB sources, including established cell lines (genetically manipulated or not), PDCLs or biopsies (fresh or pre-cultured) [49]. Most xenografts are orthotopic, in which the brain TME with its own cellular and extracellular components, organ architectures and anatomical barriers are the natural ones for grafted GB cells, mimicking the GB progression occurring inside the patient [49].

##### Mouse Xenografts

Experimental GB mouse models can be rapidly generated thorough GB cell injection at both the embryonic or post-natal stages [36,140,141]. The origin of implanted human cells and the site of implantation are key conditions which determine the features of the developing tumors in terms of genetic and phenotypic similarity to human GB. An experimental mouse heterograft is simpler to perform, via a subcutaneous injection, and to follow over time (tumor mass can be directly measured being the tumor easily accessible through the skin). Ortho transplantation, instead, requires a stereotactical intracranial injection; therefore, it is more invasive for the animal, with a higher risk of complications. Engrafted GB cells can be established cell lines or PDXs. Cell lines, due to spending a long time in culture, can be very dissimilar from the original GB tumor, and may lack some GB features, such as the infiltrative potential. The PDX mouse model involves transplanting patient-derived tumor cells, either directly or after in vitro culture, even though genetic modification may occur in the latter case, causing the tumor cells to diverge from the original explanted tumor [72,142]. The past few decades have been dominated by heterotopic graft models in which GB cell lines were injected subcutaneously into mice. Primary xenograft GB lines were established and maintained thorough direct implantation of GB biopsies heterotopically into the flank of nude mice and subsequent serial passage of these tumors in the flank of mice [140]. However, the most representative GB graft model is the patient-derived orthotopic xenograft (PDOX), in which GB primary cells are injected into the brain, providing the natural stromal support and tumor environment. An adopted strategy was to maintain GB PDXs by serial subcutaneous passaging in nude mice and then use them to generate a panel of GB PDOXs. A library of PDOX models using surgical GB samples has been also established, recapitulating biological and histopathological features of human GB in situ [143]. The use of tumor fragments, without passage in culture, has the great advantage of preserving the genetic and phenotypic features of the tumor of origin, but shows also important technical limitations, such as an extremely variable success rate, especially using small tumor fragments, and a long tumor latency [142]. Possible compromise might be obtained by intermediate steps, such as the derivation of gliomaspheres in vitro, which, when injected in a mouse brain, form tumors with a high efficiency and speed, even though these passages can lower the similarity with the tumor of origin.

Immunocompromised mouse strains, such as athymic nude mice, severe combined immunodeficient (SCID) mice, non-obese diabetic (NOD) mice or NOD-SCID mice are usually used in order to avoid immune rejection of grafted cells [36]. The use of immunodeficient mice, however, enables the study of the interactions between grafted human GB cells and the immune system as well as evaluation of candidate immunotherapies; for this reason, different approaches have been developed to use also immunocompetent mice. Hoffmann and coauthors took advantage of the immune-privileged developmental time window to successfully inject human GB cells into the telencephalic ventricle of wild-type embryos at 12.5 days of development [141]. As an alternative approach, GB humanized mice have been obtained by engrafting into immunodeficient mice human stromal/hematopoietic stem cells which then differentiate into various immune cell types [144]. An inbred strain of immunocompetent mice was finally used to obtain an orthotopic xenotransplant model of human GB cells [145,146].

##### Zebrafish Xenografts

Xenografted human GB cells or PDXs can also be injected into zebrafish models. Using zebrafish as model systems has several advantages, including animal small size, high fecundity, ex utero development and cost-efficient husbandry. Microinjection is usually performed at the embryonic stage, when larvae are transparent and easily accessible, and, as with mouse embryos, immune surveillance has not yet developed [147]. Microtumors form in three or four days, compared to the weeks necessary in rodents. Moreover, the use of fluorescently labelled cells renders in vivo imaging easy to perform [148,149]. The first attempts involved transplantation of adherent GB cell lines into the yolk sac, demonstrating the formation of tumors and angiogenesis, as well as allowing for the study of response to treatments [147,150,151]. Further studies xenotrasplanted conventional GB cell lines orthotopically into zebrafish larva brain to analyze tumor invasion, angiogenesis and the impact of Wnt signaling during GB cell differentiation [152,153]. This approach was technically challenging due to the high lethality of transplanted embryos and has been standardized to have more reliable and reproducible results to follow tumor growth and invasion with real-time imaging [154]. Recently, novel, simpler methods have been developed to overcome intracranial transplantation of GB cells into single zebrafish embryos based on cell grafting at blastula stage. These methods allow for easy, fast and automatable orthotopic injection, opening the way to high-throughput drug screenings [155].

In addition to wild-type zebrafish, transgenic strains were also used to simplify and fast the in vivo analysis. Among them, Casper mutants, which remain transparent into adulthood due to the inhibition of melanophore formation and Tg (fli1:EGFP), in which enhanced green fluorescent protein (EGFP) marks the endogenous vasculature, commonly utilized for research on vasculogenesis, angiogenesis, and the development of metastases [149]. U87 cells were often xenografted in GB angiogenesis studies, whereas U251 cells in GB proliferation studies, and PDXs were employed for preclinical studies with clinical relevance. In conclusion, zebrafish can be an excellent model for large-scale drug discovery, and their predictive ability has been harnessed in multiple high-throughput screening studies.

#### 5.2.2. Purpose, Strengths and Limitations

Until now, mouse orthotopic xenograft models have been widely used to study drug performance in an in vivo context, even though the use of immunodeficient mice to suppress host immune rejection enables the study of human physiological immune responses as well as of the impact of immunotherapies. Zebrafish xenograft models are a valid alternative to mouse due to several advantages, including fast tumor formation, high number, rapid development and the small size of the offspring, which make them suitable for implementation in high-throughput screening contexts [69]. Zebrafish graft models are used to study a plethora of aspects of the tumorigenesis process, including cell proliferation, migration, angiogenesis, tumor invasion and BBB permeability [148,156,157]. However, animal xenograft models also have important limitations, in addition to obvious ethical concerns. Despite similarities in the overall cellular architecture, humans have a more complex and evolved neocortex and stark differences in gene expression patterns [59], especially those of non-neuronal cells, such as microglia, which are thought to play a key role in GB progression [60,61]. Ultimately, brain tumors are not cell-autonomous, and they can be influenced by the host brain environment [158], thus decreasing the reliability and predictive ability of animal models. At present, the efficacy of candidate GB therapeutics has to be assessed in physiologically relevant systems, such as laboratory animals, which allow for the study of drug permeation in the brain [142]. However, the sudden increase in GB-on-a-chip models has the potential to significantly reproduce parental GB and transform drug development by reducing the need for animal testing and improving translation to the clinic [139,159].

### 5.3. In Silico Modeling

#### 5.3.1. Origin and Characteristics

In vitro, ex vivo and animal tumor models demand significant effort and time, and they yield a substantial amount of data that need to be systematically analyzed and understood to be meaningful in a clinical context. Noteworthily, mathematical and computational models can be employed to help in depicting the intricate progression of tumors, as they can handle the multiscale aspects of the biological processes as well as incorporate data from various imaging technologies, clinical assessments and biological experiments [49,160,161,162]. Remarkably, predictions from models can also lead to the design of targeted experiments to confirm incoming hypotheses [49]. Based on the scale at which the tumor is represented, mathematical oncology models can be divided into discrete or stochastic models, and continuum or analytical ones. The discrete models simulate the behavior of individual cancer cells, capturing their interactions with the microenvironment and the randomness of cellular events. In 1992, Dutching and coworkers published one of the first discrete developed computer models describing 3D cell growth inside tumor spheroids [163]. This approach is more appropriate for detailing in vitro studies and tumors of a small size [49,164]. On the other hand, continuum models are used to depict tumors at the tissue level, approximating tumor cells and the tumor microenvironment as continuous variables and describing the collective, averaged behavior of tumor cells. These models can accurate describe patient tumors and their response to therapy, and potentially have a high degree of clinical relevance [49,164]. Among the first continuum models were those developed in the early 2000s by Swanson and coauthors to describe the spatio-temporal growth, diffusion and invasion of GB cells into human brain [165,166].

#### 5.3.2. Purpose, Strengths and Limitations

The use of computational simulations allows us to model tumor biology in order to understand tumor disease progression, as well as molecular and functional dynamics. In silico modeling can also be used to predict treatment response and therapeutic outcomes, and, eventually, to guide clinical decisions [49,164]. Several mathematical models have been developed to describe GB progression and its main features, such as tumor heterogeneity, invasiveness, irregular angiogenesis, and also treatment response [162,167,168,169,170,171,172]. Since they are defined as such, in silico models lack experimental errors, are affordable, precise, non-invasive and usually save time by providing a fast method to systematically analyze the influence of various cellular components in diverse environmental conditions. Though aimed at patients, the majority of in silico models tend to be either theoretical or generic, as they require data that are impossible to obtain at the appropriate spatial and temporal scales for proper patient-specific parametrization. Mathematical models are typically parameterized by incorporating pre-existing data from the literature. Although recent efforts are guiding research toward this goal, the challenge persists in identifying a plausible mechanistic model and properly parametrizing it to quantitatively account for a large dataset not involved in calibration and to forecast clinical or experimental results. Regardless of the mathematical method used, however, biological data are crucial for parameterizing and validating all computational models to ensure they accurately reflect biological processes [49,115,164]. As an example, studies combining mathematical modeling and experimental results emerged, describing GB cell radially symmetric migration and invasiveness, based on data collected from U87 2D in vitro culture dispersion and a U87 tumor spheroid 3D model, respectively [173,174]. Over time, more and more sophisticated approaches integrating wet data and in silico modeling have been developed to predict treatment responses [175,176,177] or the evolutionary dynamics guiding tumor growth [178]. Mathematical models have been often used to fit data obtained by using GB microfluidic devices [179]. Finally, a new paradigm is rising in Simulation-Based Engineering and Sciences that aims to combine mathematical models with a Machine Learning approach, which can take advantage of the enormous amount of data produced by GB experimental research [179].

## 6. Future Perspectives and Conclusions

Nowadays, it is clear that different GB models have their own peculiarities, ethical concerns, strengths, weaknesses and applications. Therefore, no single model or specific model combination can be considered the ideal choice for every study purpose. Moreover, improving the reliability and predictability of GB models is essential for achieving successful clinical outcomes. To increase the translational relevance of preclinical studies, we propose a roadmap that addresses the most relevant issues starting with the easiest and working up to the long-term ones.

1. Choice of the most suitable GB model(s)

Due to the increasing number of available GB models, related protocols and strategies, it is crucial to choose the best model or set of models to study in order to guarantee the high reliability and reproducibility of results, as well as increasing the chances of therapeutic success in clinical studies. Various factors must be considered, including the model’s performance, complexity, stability and reproducibility. Other relevant practical criteria include the management of time, human resources and financial investment, the presence of the necessary expertise and/or collaborations, and the number of therapeutic agents to be tested, as well as the amount of drug needed for the specific model, a factor which can sometimes be extremely important. The weight of each criterion can differ from case to case, depending on the tumor feature being investigated and the purpose of the study. The choice of which model(s) to use, as well as the utilization pipeline, require a careful evaluation of the cost–benefit balance to avoid wasting of resources and achieving of poor outcomes. Using an efficient, customizable and easy-to-use tool, such as specific multi-criteria decision-making (MCDM) matrices, which have been employed in various contexts within the life sciences in both basic and applied research, refs. [180,181,182,183] may also be helpful. The MDCM approach enables different alternatives to be identified and compared through well-defined assessment criteria and specific metrics, providing a basis for informed evaluation and decision-making [184]. Assessment criteria can be both qualitative or quantitative and can be eventually divided into assessment sub-criteria. Examples of assessment criteria and sub-criteria could be (i) model representativity in terms of similarity with patient GB (sub-criteria: genetic mutations, cellular features, presence of ECM and stromal component, presence of BBB); (ii) model reproducibility (level of standardization, model variability, variance and reproducibility of the results); (iii) time (sub-criteria: time of generation, time of analysis; (iv) resources (sub-criteria: model availability, internal competence, collaborations, technologies/instruments). Then, depending on the metrics used and their respective acceptance ranges, a score (usually ranging from 0 to 3) is assigned to each criterion/sub-criterion. For example, for the time taken to generate the model, a score of 0 could be assigned for a timeframe of more than one year, a score of 1 for a timeframe of between six months and one year, a score of 2 for a timeframe of between three and six months, and a score of 3 for a timeframe of less than three months. Interestingly, an importance weight (usually from 1 to 3) must be assigned to each criterion/sub-criterion according to its relevance to the specific study/team. Ultimately, a final ranking of the GB models can be obtained, in which the ranking of each one is calculated by summing the final scores for each criterion/sub-criterion, which are the product of the obtained score and the importance weight. Defining the ranges associated with a specific score, as well as assigning the importance weights, allows for personalization and customization to specific needs.

2. Identification of guidelines and standardization of procedures for model usage

Optimizing, standardizing and sharing procedures is fundamental to ensuring the reliability and reproducibility of preclinical models and relative results. Currently, raising awareness among the European scientific community and various stakeholders regarding standards, data reliability and reproducibility is encouraging collaborative efforts to identify and share good practices and standard operating procedures (SOPs) [185]. Several international scientific societies and networks have initiated discussions to establish their own standards and encourage adherence to these within their respective communities [186,187,188]. This is particularly challenging in the case of GB and, more generally, tumor preclinical models for two reasons. Firstly, this research is becoming increasingly transdisciplinary, based on the integration of novel expertise and complex technologies from various disciplines, for which standardized protocols must be defined [31,139]. Secondly, the outcome of preclinical research is the identification of novel diagnostic or therapeutic solutions to be translated into clinical practice. The reliability of the outcomes, the standardization of the procedures and the accuracy of clinical predictions are all fundamental to fostering effective and efficient clinical translation, as well as compliance with related regulatory systems. Therefore, we strongly advocate a collective effort within the GB community to identify and promote initiatives in this direction. Possible initiatives include (i) the development and maintenance of publicly available repositories of validated SOPs for common experimental techniques in GB research (e.g., cellular assays, in vivo tumor modeling, imaging protocols, and ex vivo modeling); (ii) the development of training programs, workshops, and e-learning resources focused on best practices in GB experimental modeling, data analysis, and standards reporting; and (iii) the definition of quality standards and relative quality assurance programs where laboratories can undergo voluntary accreditation for adhering to established best practices and reproducibility guidelines. Finally, integration with Regulatory Frameworks would be beneficial to ensure that preclinical models and data meet the evolving requirements for translational and clinical development pathways.

3. Open platforms and integrations of the different models.

A conscious and well-designed strategy that integrates different approaches can help to discover and analyze the most effective anti-GB therapies, as well as generating more comprehensive and reliable preclinical data. The generation of a set of validated reference models or assays that can be used across laboratories and the organization of multi-center replication studies to assess reproducibility of key findings would be extremely relevant to ensure comparability of results and benchmark novel interventions. In this context, biobanks collecting GPDCLs or GBOs [75,76,101] are undoubtedly a valuable resource. We auspicate the creation of more and more centralized, open-access biobanks and data repositories for patient-derived GB models, such as organoids, xenografts, and cell lines, along with detailed metadata and methodological annotations. Furthermore, comparative analyses of outcomes collected using different GB models to investigate specific GB features and the therapeutic effects of different drugs, as in the work of Truong and coauthors [134], are also extremely valuable contributions. Finally, implementing open platforms and resources that focus on optimizing, validating and sharing increasingly sophisticated technologies, such as GB-on-a-chip, is fundamental to enhancing the use of these cutting-edge models and fostering preclinical GB research, drug discovery and validation.

4. Development of novel multimodal integrated approach

The integration of various technologies and strategies can greatly enhance our understanding of GB etiology and transform drug development by minimizing animal testing and improving clinical translation. Microfluidics, bioprinting and bioengineering are converging to create more sophisticated yet simple and reproducible GB-on-a-chip systems that address GB pathophysiology and therapeutic treatment. To this end, creating transdisciplinary networks and projects that address the issue from different theoretical and methodological perspectives is fundamental, as is establishing dedicated funding programmes. As an example, the Glioblastoma Therapeutics Network (GTN) funded by the National Cancer Institute (NCI) moves in this direction, aiming at developing novel effective therapeutics for the treatment of adult GB by in an integrated team setting to move from preclinical development to pilot clinical studies [189].

Last, but not least, strict quality control of the input data and variables is essential to ensure the reliability and reproducibility of the outcome. This is particularly important when using complex, heterogeneous model systems, either ex vivo or in vivo, as any significant difference in the input variables may be amplified. The production, isolation, collection, quantification and storage of candidate drugs for preclinical research must also be carefully standardized, validated and monitored through a quality assurance process.

The continued development and improvement of suitable GB models, alongside the fostering of a culture of openness, collaboration, and methodological rigor, and the availability and utilization of open modeling system platforms will undoubtedly increase our basic knowledge of GB pathophysiology and significantly strengthen the quality, impact, and translational relevance of GB preclinical research. We auspicate stakeholders across academia, industry and funding bodies to actively support and participate in these efforts in order to advance the field of GB treatment.

## Figures and Tables

**Table 1:** Main glioblastoma models of animal origin.

Origin	Mouse vs. Rat	Model Type	Generation	Effort		Representativity Respect to Patient GB	Complexity			Purpose	Throughput
				Cost	Time		ECM	TME	BBB	Applications	
**Rat**	**Strengths** (i) Larger brain size facilitating stereotactic implantation (ii) Larger tumor size improving in vivo imaging and allowing higher drug dose administration	*Chemically induced mutants*	Mutagen injections	H	Variable from few to several weeks	Variable Different tumor mechanisms and brain architecture	+	+	+	Response to different therapies; Immune mechanisms	L
		*Cell lines*	Derived from in vivo mutants	L	Weeks	L/M Can differ from patient GB both genetically and phenotypically; Accumulate genetic mutations in culture	-	-	-	Cancer cell features; Response to therapy	H
	**Weaknesses** (i) Cannot be easily genetically manipulated (ii) More expensive to purchase and maintain (iii) Minor availability of specific reagents										
		*Syngeneic grafts*	Intracranial or intravenous injection of cell lines	M	Variable from few to several weeks	Variable Different tumor mechanisms and brain architecture; Depending also on transplanted cell features	+	+	+	Response to therapies; Immune mechanisms	M
**Mouse**	**Strengths** (i) Possibility to be genetically manipulated to harbor specific mutations (ii) Smaller and easier to maintain (iii) Major availability for specific reagents	*Chemically induced mutants*	Mutagen injections	M/H	Variable from few to several weeks	Variable Different tumor mechanisms and brain architecture	+	+	+	Response to different therapies; Immune mechanisms	L
		*Genetically engineered* *mutants*	Cre/loxP	M/H	10–14 months	Variable Different tumor mechanisms and brain architecture; Carrying selected targeted mutations by origin cannot replicate the GB heterogeneity	+	+	+	Cancer Biology; Effect of specific gene mutation; Response to therapy; Immune mechanisms	L/M
			Transposon induced		6–8 months						
	**Weaknesses** (i) Smaller animal size complicating stereotactic procedures (ii) Smaller tumor size complicating in vivo imaging and treatment evaluation		CRISPR/ Cas9		5–7 months						
			Engineered virus induced		Weeks						
		*Cell lines*	Derived from in vivo mutants	L	weeks	L/M Can differ from patient GB both genetically and phenotypically; Accumulate genetic mutations in culture	-	-	-	Cancer cell features; Response to therapy	H
		*Syngeneic Grafts*	Intracranial or intravenous injection of cell lines	M	Variable from few to several weeks	Variable Different tumor mechanisms and brain architecture; Depending also on transplanted cell features	+	+	+	Response to different therapies; Immune mechanisms	M

Abbreviations: BBB, blood–brain barrier; ECM, extracellular matrix; H, high; L, low; M, medium; TME, tumor microenvironment.

**Table 3:** Main glioblastoma modeling strategies.

Modeling Strategy		Generation	Strengths	Weaknesses	Purpose/Applications						
					CP/D	CM/I	DA	RT	TME	BBB	T
2D	Monolayer Culture	Culture of adherent cells in medium with serum and nutrients	Easy; Cost effective; High availability of standardized protocols and commercial reagents	Subject to genetic drift; Not reproducing the spatial complexity and intricate cell relationships of in vivo GB	+	+	+	+			H
3D	Spheroid	Spontaneous aggregation in suspension									
	*-MCTS*	Mainly GB cell lines	Easy to culture and genetically manipulate	Low histological similarity to in vivo GB	+	+	+	+			M/H
	*-Gliomasphere*	GB primary cells	Representing genetic and phenotypic in vivo GB heterogeneity; Suitable for personalized medicine	Low architecture complexity respect to in vivo GB	+	+	+	+			L/M
	*-OMS*	Tumor and not tumor primary cells	More similar to in vivo GB; Reproducing TME; Suitable for personalized medicine	Low architecture complexity respect to in vivo GB	+	+	+	+	+		L/M
	GB Organoid	Free self-assembly of GB primary cells	High complexity; Representing heterogeneity of in vivo GB; Suitable for personalized medicine; Versatile and customizable	Low control and reproducibility	+	+	+	+	+/-		M
	GLICO	Incorporation of primary GSCs into brain organoids	High correlation with in vivo GB; Reproducing interaction with TME; Suitable for personalized medicine	High cost, Low control	+	+		+	+		L
	Scaffold	Embedding and growth of primary GB cells on defined scaffolds	Representing heterogeneity of in vivo GB; Reproducible; Suitable for personalized medicine	Scaffold biocompatibility	+	+	+	+			M
	Bioprinting	Combination of GB cells (and eventually TME cells) in a bioreactor with a bioink	Highly controlled; Reproducible; Depending on the types of bioprinted cells can highly represent GB complexity and TME	High cost; High specialization; Need of suitable bioinks to mimic TME complexity	+	+	+	+	+/-	+/-	M
	Microfluidic	Very small volumes of cells and fluids are combined for perfusion culturing	Low amounts of cells and materials; Reproducible; Dynamic environments closer resembling in vivo GB; Depending on the types of cells can highly represent GB complexity and TME	High cost and specialization; Need of performant materials to build the devices; Challenging downstream sample analysis	+	+	+	+	+/-	+/-	M/H
	GB-on-a-chip	Integration of different technologies in a chip	Controlled; Reproducible; Highly resembling in vivo GB; Suitable for personalized medicine	High cost and specialization; Need of performant materials to build the devices; Challenging downstream sample analysis	+	+	+	+	+	+/-	M
In vivo	Mouse xenograft	Injection of GB cells (cell lines, GPDCs or GB tumor pieces)									L/M
	*-Heterotopic*	Intravenous	Simple; High efficiency	High costs of housing for immunodeficient mice; Different tumor environment	+		+	+			L/M
	*-Orthotopic*	Intracranial	Representative of the in vivo physiological TME	High costs of housing for immunodeficient mice; Complex; High mortality	+	+		+	+	+	L/M
	Zebrafish xenograft	Injection of GB cells (cell lines, GPDCs or GB tumor pieces)									M/H
	*-Heterotopic*	Intra yolk sac	Simple; High efficiency; Low cost of housing; Transparency; In vivo imaging	Different tumor environment	+		+	+			M/H
	*-Orthotopic*	Intracranial or into the blastula (injected cells incorporate into the brain)	Low cost of housing; Representative of the in vivo physiological TME; In vivo imaging; Transparency	Complex	+	+	+	+	+	+	M
In silico	Discrete Model	Simulation at single cell level	Can accurately describe the behavior of single cells in simpler contexts	Need of experimental data to create, validate and optimize the model	+	+			+		M/H
	Continuum Model	Simulation at tissue level	Can accurately describe patient GB		+	+		+	+	+	M/H

Abbreviations: BBB, blood–brain barrier; CM, cell migration; CP/D, cell proliferation and death; DA, drug assays; GLICO, Cerebral organoid Glioma; GB, Glioblastoma; GPDCs, Glioblastoma patient derived cells; GSCs, Glioblastoma stem-like cells; H, high; L, low; M, medium; MCTS, multicellular tumor spheroids; OMS, organotypic multicellular spheroids; RT, resistance to therapy; T, throughput; TME, tumor microenvironment.

## Data Availability

No data were created.

## Abbreviations

The following abbreviations are used in this manuscript:

2D	Two-Dimensional
3D	Three-Dimensional
AKT	Protein Kinase B
BBB	Blood–Brain Barrier
bFGF	basic Fibroblast Growth actor
BRCA1	BReast CAncer gene 1
BTB	Blood-Tumor Barrier
CAR-T	Chimeric Antigen Receptor T
ECM	ExtraCellular Matrix
EGFR	Epidermal Growth Factor Receptor
EGFP	Enhanced Green Fluorescent Protein
EMT	Epithelial Mesenchymal Transition
ENU	Ethylnitrosourea
GAMs	Glioblastoma-associated macrophages
GB	Glioblastoma
GBO	Glioblastoma Organoid
GTN	Glioblastoma Therapeutics Network
GCO	Glioblastoma Cortical Organoid
GCOA	Glioblastoma-Cerebral Organoid Assembloid
GEMMs	Genetically Engineered Mouse Models
GLICO	Cerebral Organoid Glioma
GPDCs	Glioblastoma Patient-Derived Cells
GPDCL	Glioblastoma Patient-Derived Cells lines
GSCs	Glioblastoma Stem Cells
H	High
hiPSCs	human-induced Pluripotent Stem Cells
JAK/STAT	Janus Kinase/Signal Transducer and Activator of Transcription
L	Low
M	Medium
MCDM	Multi-Criteria Decision-Making
MCTS	Multicellular Tumor Spheroids
MGMT	O-6-methylguanine-DNA methyltransferase
MNU	Methylnitrosourea
mTOR	Mechanistic Target of Rapamycin
NCI	National Cancer Institute
NOD	Non-Obese Diabetic
OMS	Organotypic Multicellular Spheroids
PDCLs	Patient-Derived Cells lines
PDGF	Platelet-Derived Growth Factor
PDGFR	Platelet-Derived Growth Factor Receptor
PDMS	PolyDiMethylSiloxane
PDOX	Patient-Derived Orthotopic Xenograft
PDX	Patient-Derived Xenograft
PI3K	Phosphatidylinositol 3-Kinase
SCID	Severe Combined ImmunoDeficient
SOPs	Standard Operating Procedures
TME	Tumor MicroEnvironment
TMZ	Temozolomide
VEGF	Vascular Endothelial Growth Factor
